# Rapid screening of point mutations by mismatch amplification mutation assay PCR

**DOI:** 10.1007/s00253-024-13036-2

**Published:** 2024-02-02

**Authors:** Feng Zhang, Zhen Yang Liu, Shuai Liu, Wei Guo Zhang, Bing Bing Wang, Chang Lon Li, Jian Zhong Xu

**Affiliations:** The Key Laboratory of Industrial Biotechnology, Ministry of Education, School of Biotechnology, Jiangnan University, 1800 Lihu Road, Wuxi, 214122 People’s Republic of China

**Keywords:** *Corynebacterium glutamicum*, Genome editing, Point mutations, MAMA PCR, Mutation libraries

## Abstract

**Abstract:**

Metabolic engineering frequently makes use of point mutation and saturation mutation library creation. At present, sequencing is the only reliable and direct technique to detect point mutation and screen saturation mutation library. In this study, mismatch amplification mutation assay (MAMA) PCR was used to detect point mutation and screen saturation mutation library. In order to fine-tune the expression of *odhA* encoding 2-oxoglutarate dehydrogenase E1 component, a saturating mutant library of the RBS of *odhA* was created in *Corynebacterium glutamicum* P12 based on the CRISPR-Cas2a genome editing system, which increased the l-proline production by 81.3%. MAMA PCR was used to filter out 42% of the non-mutant transformants in the mutant library, which effectively reduced the workload of the subsequent fermentation test and the number of sequenced samples. The rapid and sensitive MAMA-PCR method established in this study provides a general strategy for detecting point mutations and improving the efficiency of mutation library screening.

**Key points:**

*• MAMA PCR was optimized and developed to detect point mutation.*

*• MAMA PCR greatly improves the screening efficiency of point mutation.*

*• Attenuation of odhA expression in P12 effectively improves proline production.*

**Supplementary Information:**

The online version contains supplementary material available at 10.1007/s00253-024-13036-2.

## Introduction

Enzyme catalysis, biotransformation, and microbial fermentation have emerged as crucial processes for producing a variety of high value–added products (Choi et al. 2019; Liu et al. 2020; Wen and Bao 2021). However, in order to develop efficient chassis cells, microbial fermentation and biotransformation necessarily need to change important enzyme genes in order to boost enzyme activity (Gao et al. 2022; Liu et al. 2020; Thu Ho et al. 2013), relieve the feedback inhibition of the product (Liu et al. 2022a; Vogt et al. 2014), or modify gene expression (Wen and Bao 2019; Zhang et al. 2020), which is advantageous to product synthesis. The approach of introducing point mutation into the coding gene is frequently used to improve enzyme performance. Point mutagenesis includes just a few base substitutions as opposed to the knocking out and insertion of DNA fragment; therefore, it is sometimes challenging to recognize mutations by the size of PCR product fragments. Therefore, gene sequencing is a common method to identify whether transformants are mutated. Although a variety of editing methods have been established at present, even an efficient genome-editing system based on CRISPR can hardly guarantee the editing efficiency of each site to reach 100% (Doench et al. 2014; Jiang et al. 2017; Kim et al. 2018; Wang et al. 2021a, b; Zhang et al. 2020). As a result, when the editing efficiency is low, the majority of the transformants are false positives, making it necessary to sequence more samples in order to screen for mutants, which increases the cost of sequencing. Therefore, it is necessary to construct an efficient and low-cost method to detect point mutations.

**Fig. 1:**
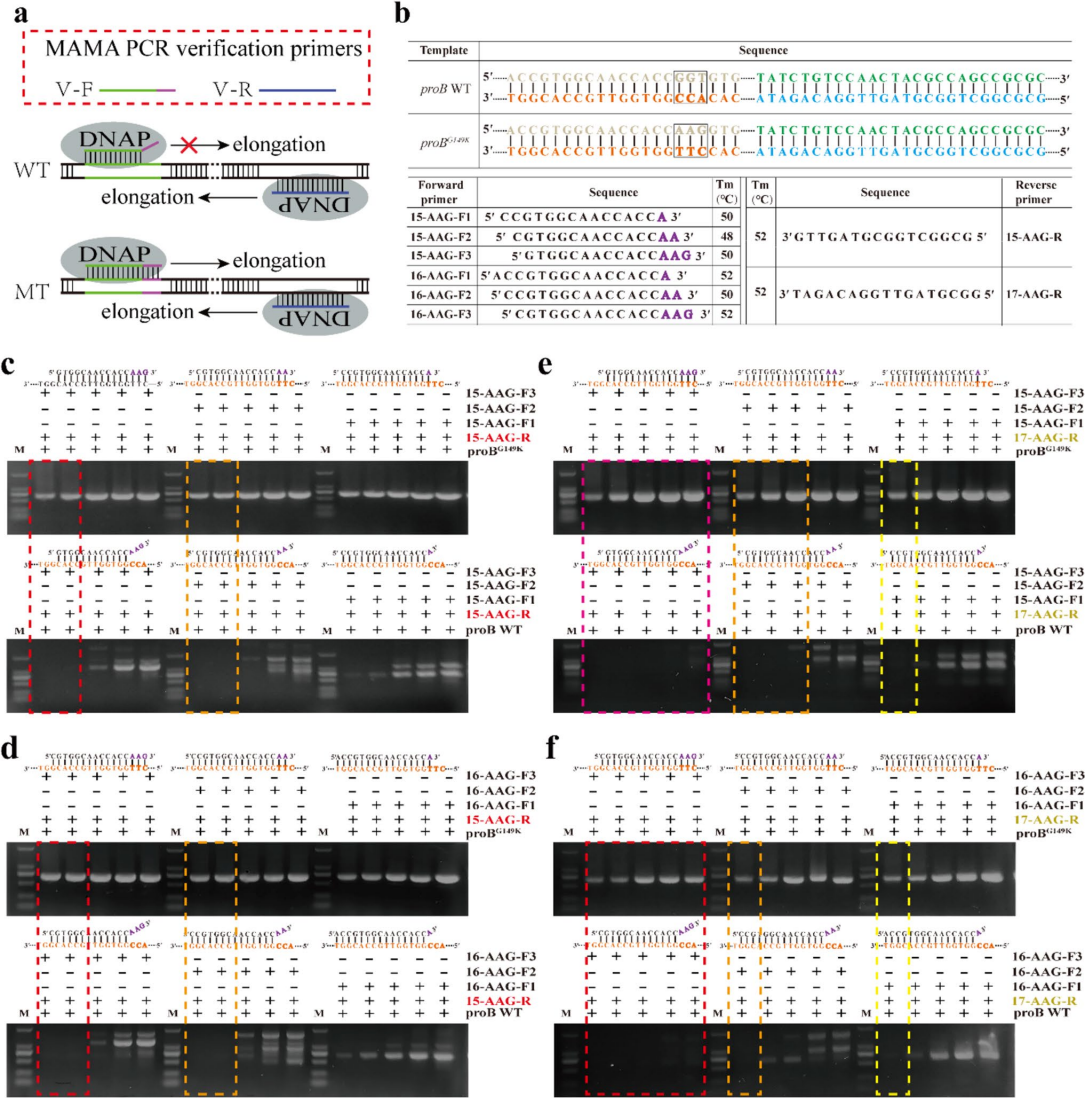
Base substitutions at the *CgproB* site were identified using PCR. **a** Schematic diagram of the principle of Taq polymerase–based PCR to identify point mutations. The 3′-OH of the oligonucleotide primer is a necessary condition for DNA polymerase to initiate polymerase chain reaction. Taq polymerase does not have 3′-5′ proofreading activity. Therefore, a base mismatch is introduced at the 3′ end of the primer. It will cause the polymerase chain reaction not to be initiated, so the mutant strain and the wild-type strain can be identified by PCR. **b** Schematic diagram of primers of different lengths and the number of mismatched bases at the 3′ end. Used to examine the effect of different base mismatch numbers and primers of different lengths (increasing the **△**TM value of upstream and downstream primers) on PCR. **c–f** When there is a 1–3 bp base mismatch at the 3′ end of the primer, the base substitution can be effectively identified as the temperature increases and the difference in the TM value of the primer becomes larger

Compared with sequencing, PCR is a rapid and low-cost method to identify mutations. PCR requires the participation of primers, and the hydroxyl group at the 3′ terminal of primers is necessary (Tindall 1988). Due to the lack of 3′-5′ exonucleolytic proofreading activity in Taq polymerase, the mismatch between the primer’s 3′ terminal base and the template will stop the PCR (Tindall 1988). Given this, MAMA PCR was well established and experimented in the late 1980s in the detection of point mutation of several disease conditions (Cui et al. 2016; Deekshit et al. 2019; Santhosh et al. 2017). The MAMA PCR technique is also widely used for the detection of point mutations in the quinolone resistance determining regions (QRDRs) of fluoroquinolone-resistant bacterial pathogens (Deekshit et al. 2019; Kakuta et al. 2020; Ota et al. 2022). The basis of the technique is dependent on primer designing. Taq DNA polymerase is unable to perform the extension process because of a single nucleotide mismatch at the forward oligonucleotide primer’s 3′ proximity. Thus, the primers produce a PCR fragment in the mutant harboring point mutation, whereas it is not possible to yield a product with a wild type at the site covered by the mismatch positions on the MAMA PCR primer from any gene (Fig. 1a). Thus, MAMA PCR is a promising method to identify point mutations. However, MAMA PCR is affected by the base composition and the number of bases at the terminal of mismatched primer (Kwok et al. 1990). This is unfavorable for us to use MAMA PCR to identify point mutations more generally. Therefore, how to effectively apply MAMA PCR to identify point mutations is still a problem to be solved.

*Corynebacterium glutamicum*, a high-GC content, Gram-positive soil bacterium, is an important industrial microorganism for food safety, which is used to produce a variety of amino acids (Dele-Osibanjo et al. 2019; Ghiffary et al. 2022; Liu et al. 2022b; Yu et al. 2021). Although l-proline is one of nonessential amino acids for organisms, it has been widely used in agriculture, feed industry, food industry, and pharmaceutical industry (Jensen 2013; Ren et al. 2020; Zhang et al. 2020, 2017). Based on metabolic engineering, significant progress has been achieved lately in engineering *C. glutamicum* to produce l-proline (Liu et al. 2022a; Zhang et al. 2020). In this study, we optimized the MAMA PCR technique in *C. glutamicum*, enabling it to detect point mutations and even single base substitutions. We found that by adjusting the paired primers and elevating the annealing temperature of PCR, MAMA PCR can rapidly and accurately distinguish between wild-type and mutant templates in addition to increasing the number of mismatched bases. Moreover, we applied MAMA PCR to the preliminary screening of mutant library. It is reported that weakening the expression of *odhA* can greatly promote the synthesis of l-glutamate and amino acids with l-glutamate as precursor (Wen and Bao 2019; Zhang et al. 2020). However, excessive inhibition of OdhA activity will impair cell growth; hence, fine-tuning *odhA* expression can give consideration to cell growth and l-glutamate synthesis. In order to fine-tune the expression of *odhA*, a saturating mutant library of the RBS of *odhA* was created in proline-producing strain *C. glutamicum* P12 based on the CRISPR-Cas2a genome editing system, which increased the l-proline production by 81.3%. MAMA PCR was used to filter out 42% of the non-mutant transformants in the mutant library, which effectively reduced the workload of the subsequent fermentation test and the number of sequenced samples. In a word, we provide an efficient method to identify point mutation and screen point mutation library. Although this method has been tested in *C. glutamicum*, it is also applicable in other microorganisms.

## Materials and methods

### Strains, medium, and culture conditions

Strains used in this study are listed in Table 1. *E. coli* JM109 used for plasmid cloning were aerobically cultivated at 37 °C in Luria–Bertani (LB) broth. Appropriately, kanamycin (Kan, 50 μg/mL) or chloramphenicol (Cm. 20 μg/mL) was added to the medium. *C. glutamicum* and the derivatives were aerobically cultivated at 30 °C in LBG medium (LB medium supplemented with 5 g/L glucose). The Epo medium (LBG medium supplemented with 3% glycine, 0.1% Tween 80, and 0.4% isoniazid) used for growing electroporation competent cells and the LBHIS (2.5-g/L yeast extract, 5-g/L tryptone, 5-g/L NaCl, 18.5-g/L Brain Heart Infusion powder, and 91-g/L sorbitol) plates used for obtaining transformants of *C. glutamicum* were prepared as previously described (Xu 2014). The LBS medium (LB medium supplemented with 10% sucrose) is used for selection of double crossover *C. glutamicum*. Spectinomycin (50 μg/mL), Kan (25 μg/mL), and Cm (5 μg/mL) or isopropyl-β-d-thiogalactoside (IPTG, 0.01 mM) was added to LBHIS medium as required.

**Table 1:** Strains and plasmids used in this study

Strain	Relevant characteristic(s)	Reference
*E. coli* JM109	recA1, supE44 end1 hsdR17 (r − k, m + k) gyrA96 relA1 thi (lac-proAB) F’ [traD36 *proAB* + *lacI*q *lacZ*ΔM15]	Takara
*C. glutamicum* ATCC 13032	Type strain	ATCC
△LtbR	*C. glutamicum* XQ-9 derivative with in-frame deletion of *ltbR*	(Wang et al. 2019b)
△LtbRAHAIR^M^	∆LtbR derivative with chromosomally integrated mutations into *ilvC* coding for amino acid exchanges S34G, L48E, and R49F	(Wang et al. 2019b)
13032proB^G149K^	*C. glutamicum* ATCC 13032 derivative with chromosomally integrated mutations into *proB* coding for amino acid exchanges G149K	This study
13032zwf^A243T^	*C. glutamicum* ATCC 13032 derivative with chromosomally integrated mutations into *zwf* coding for amino acid exchanges A243T	This study
13032gnd^S361F^	*C. glutamicum* ATCC 13032 derivative with chromosomally integrated mutations into *gnd* coding for amino acid exchanges S361F	This study
*C. glutamicum* P12	l-proline producer *C. glutamicum* strain created by random mutagenesis; resistant to succinic acid, sulfaguanidine and 3,4-dehydroproline	(Zhang 1996)
Plasmids		
pK18*mobsacB*	Kan^r^, integration vector; *oriV*_*Ec*_* oriT sacB*, allows for selection of double crossover *C. glutamicum*	(Schäfer A 1994)
pFSC	Cm^r^, *pBL1 oriV*_*C. glu.*_ pUC *oriV*_*E. coli*_, P*tac-cas9*	(Peng et al. 2017)
pFST	Kan^r^, *repA oriV*_*C. glu.*_* pMB1 oriV*_*E. coli*_, P*trc*	(Peng et al. 2017)
pJYS3_crtYf	Kan^r^, *pBL1*^*ts*^* oriV*_*C. glu.*_ pSC101 *oriV*_*E. coli*_ P_*lacM*_*-FnCpf1*, P_*j23119*_- crRNA targeting *crtYf*	(Jiang et al. 2017)
pFST-gRNA1	Kan^r^, *repA oriV*_*C. glu.*_* pMB1 oriV*_*E. coli*_, P*trc-*sgRNA1 targeting *zwf*	This study
pFST-gRNA2	Kan^r^, *repA oriV*_*C. glu.*_* pMB1 oriV*_*E. coli*_, P*trc-*sgRNA 2 targeting *zwf*	This study
pFST-gRNA4	Kan^r^, *repA oriV*_*C. glu.*_* pMB1 oriV*_*E. coli*_, P*trc-*sgRNA targeting *gnd*	This study
pJYS3_0	Kan^r^, *pBL1*^*ts*^* oriV*_*C. glu.*_* pSC101 oriV*_*E. coli*_ P_*lacM*_*-FnCpf1*	This study
pJYS3_gRNA3	Kan^r^, *pBL1*^*ts*^* oriV*_*C. glu.*_* pSC101 oriV*_*E. coli*_ P_*lacM*_*-FnCpf1*, P_*j23119*_- crRNA targeting *zwf*	This study
pJYS3_gRNA5	Kan^r^, *pBL1*^*ts*^* oriV*_*C. glu.*_* pSC101 oriV*_*E. coli*_ P_*lacM*_*-FnCpf1*, P_*j23119*_- crRNA targeting *gnd*	This study
pFST-proB^G149K^	Kan^r^, *repA oriV*_*C. glu.*_* pMB1 oriV*_*E. coli*_, P*trc-*sgRNA targeting *proB*, 0.5 kb upstream and downstream homologous arms	This study
pJYS3_odhA_RBS_-LIB	Kan^r^, *pBL1*^*ts*^* oriV*_*C. glu.*_ pSC101 *oriV*_*E. coli*_ P_*lacM*_*-FnCpf1*, P_*j23119*_- crRNA targeting *odhA*_*RBS*_, 1 kb upstream and downstream homologous arms. Different substitution bases are carried at *odhA*_*RBS*_ on the homologous template	This study
pK18-Zwf^A243T^	Kan^r^, pK18*mobsacB* derivative with chromosomally integrated mutations into *zwf* coding for amino acid exchanges A243T	This study
pK18-Gnd^S361F^	Kan^r^, pK18*mobsacB* derivative with chromosomally integrated mutations into *gnd* coding for amino acid exchanges S361F	This study

Batch cultivation in shake flasks was performed in 500-mL flasks containing 25 mL of medium A at 30 °C and 100 rpm (reciprocating incubator). Medium A contained (per liter): 160-g glucose, 40-g corn steep powder (Angel Yeast Co., Ltd), 35-g (NH_4_)_2_SO_4_, 1.0-g KH_2_SO_4_, 0.5-g MgSO_4_∙7H_2_O, 0.01-g FeSO_4_, 100-μg biotin, 100-μg thiamine-HCl, and 40-g CaCO_3_, pH7.2–7.5. The effect of glutamate on proline fermentation was tested in strain P12, and 10, 20, 30, and 40-g/L glutamate was supplemented in medium A.

### Construction of plasmids and plasmid library

The plasmids and primers used in this study are listed in Table 1 and Table S2, respectively. Chromosomal DNA was extracted from *C. glutamicum* ATCC 13032 and *C. glutamicum* P12 using an Ezup Bacterial Genomic DNA Extraction Kit (Vazyme, Nanjing, China). The target gene segments were amplified using 2 × Phanta Master Mix (Vazyme, Nanjing, China) from the appropriate DNA template. Taq polymerase (2 × Rapid Taq Master Mix (Vazyme, Nanjing, China)) was used in MAMA PCR. The plasmid construction and transformation were referred to Xu et al. (2014a, b). Recombination was conducted using the ClonExpress II and ClonExpress MultiS One Step Cloning Kit (Vazyme, Nanjing, China). Restriction endonucleases and T4 DNA ligase were purchased from TaKaRa (Dalian, China). The detailed plasmid construction process is shown in the Supplementary Methods section of Supplementary Information.

### Construction of C. glutamicum recombinant strains

The preparation of electro-competent cells for *C. glutamicum* was performed according to the method described previously with appropriate modifications (Liu et al. 2022a). Cells were cultured in 50-mL flasks with 10 ml of LBG medium for 10–13 h, and then 3-mL were transferred to 100 mL of fresh Epo medium and cultivated at 30 °C. When the ΔOD_600_ of the culture reached to 0.4–0.5, the cells were ice-bathed for 20 min and were then harvested by centrifugation at 4 °C for 5 min at 4000 rpm. After washing 3 times using 4 °C pre-chilled 10% glycerol, the cells were resuspended in 0.3 mL of 10.0% (v/v) glycerol. Plasmid was added to competent cells and transferred to a 1-mm electroporation cuvette (Bio-Rad Laboratories, Shanghai, China). Electroporation was performed using an GenePulser Xcell™ (Bio-Rad Laboratories, Shanghai, China) with parameter settings of 1800 V and 5 ms. Subsequently, 1 mL of LBHIS medium was immediately added, and the suspension was rapidly incubated at 46 °C for 6 min. The cells were incubated at 30 °C for 2 h, and then spread on LBHIS plates supplemented with antibiotics and IPTG as required and incubated at 30 °C until colonies appeared.

Construction of 13032proB^G149K^ strain was based on CRISPR-Cas9 system (Peng et al. 2017). Firstly, pFSC was transformed into *C. glutamicum* ATCC 13032 to obtain strain 13032/pFSC. Then, the plasmid pFST-proB-proB^G149K^ was transformed into *C. glutamicum* ATCC 13032/pFSC, and coated on LBHIS plate supplemented with 10 μg/LIPTG, 10-mg/L chloramphenicol, and 25-mg/L kanamycin. The 13032proB^G149K^ transformant was identified by sequencing.

Construction of 13032zwf ^A243T^ and 13032gnd ^S361F^ strain based on the traditional pK18*mobsacB*–based gene insertion were performed as previously described (Xu 2014).

### Construction of mutant library of l-proline hyper-producers and cultivation assay

Electrocompetent cells of *C. glutamicum* P12 were prepared as described above. For in vivo point saturation mutagenesis of *odhA*_*RBS*_, a plasmid library pJYS3_odhA_RBS_-LIB was used for electroporation. *C. glutamicum* P12 was transformed with 2-μg plasmid library pJYS3_odhA_RBS_-LIB. The correct transformants verified by MAMA PCR were inoculated into 48-well plates for l-proline fermentation tests. Fermentation volume was 800 ml, incubation temperature was 30 °C, rotation speed was 300 r.p.m. (THZ-C-L, QIANGLE, China), and incubation time was 72 h. Medium A was used as l-proline fermentation medium.

### Primer design and MAMA PCR protocol

**Fig. 2:**
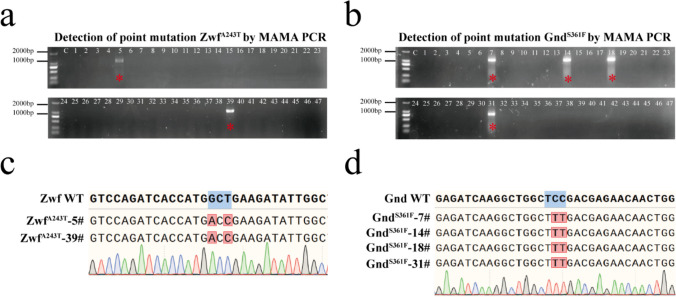
Detection of *zwf* and *gnd* mutations by MAMA PCR. **a** The mutation of Zwf^A243T^ in 47 recombinants edited by double crossover was detected by MAMA PCR. Primers Zwf^A243T^-V-F (MAMA primer) and Zwf^A243T^-V-R were used to detect Zwf^A243T^ point mutation. The annealing temperature of primers is 63.6 °C, and other PCR parameters are as described in the “Materials and Methods” section. **b** The mutation of Gnd^S361F^ in 47 recombinants edited by double crossover was detected by MAMA PCR. Gnd^S361F^-V-F (MAMA primer) and Gnd^S361F^-V-R were used to detect Gnd^S361F^ point mutation. The annealing temperature of primers is 60.9 °C, and other PCR parameters are as described in Materials and Methods. * represent that the mutation was identified in the recombinant by MAMA PCR. **(c)** Sequencing results of recombinants with identified mutations in **a**. **d** Sequencing results of recombinants with identified mutations in **b**

The rationale behind MAMA PCR is that a single nucleotide mismatch at the 3′ extremity of the annealed reverse primer renders *Taq* polymerase unable to extend the primer. So, the absence of the specific PCR product reveals a deviation from the wild-type DNA sequence. The MAMA PCR primers for point mutation ProB^G149K^ detection are shown in Fig. 1a. The MAMA primers for RBS mutation library of *odhA* gene screening are shown in Fig. 2a. In each PCR, a reverse primer and a MAMA primer were used in a PCR for mutation detection. In this study, we enhanced the 3′ mismatch effect by introducing more base mismatches into 3′ of MAMA PCR. In the PCR procedure, the 3′ mismatch effect can also be effectively enhanced by increasing the annealing temperature of primer and template. Moreover, the 3′ mismatch effect was effectively enhanced by replacing the primer paired with MAMA PCR.

### PCR experiments

The suitable annealing temperature of primers was tested by pre-amplification PCR. The annealing temperature of primers that cannot amplify PCR products is found by pre-amplification PCR, and then these temperatures are used as the annealing temperature of PCR to verify mutation. For each point mutation verification PCR program, other parameters are consistent with the pre-amplification PCR except the annealing temperature. For each pre-amplification PCR, 1μL (~ 100 ng) genomic DNA template mismatched with MAMA PCR primer was added to a final volume of 50 μL containing: 25 μL 2 × Rapid Taq Master Mix, 1μL of 10 nmol forward primer, 1μL of 10 nmol MAMA primer, and 20μL ddH2O. Pre-amplification was carried out on a DNA Thermolyne (LongGene, A300, Hangzhou, China) programmed as follows: initial denaturation at 95 °C for 10 min and 32 cycles of denaturation at 95 °C for 15 s, annealing at a temperature gradient range of 50–70 °C for 15 s and extension at 72 °C for 15 s, with a final step of 72 °C for 3 min. PCR products were visualized on horizontal 1.0% agarose gels in 1 × TAE buffer, loaded with 7μL of reaction mix and stained with GoldenView™ after electrophoresis.

### Analytical methods

Cell growth was calculated by measuring the optical density at 600 nm (OD_600_) using a spectrophotometer (Shanghai, China). Glucose and l-glutamate concentrations were determined using the SBA-40E immobilized enzyme biosensor (Shandong, China). l-proline was analyzed by reversed-phase high-pressure liquid chromatography (HPLC) using Agilent 1290 system (Agilent, Palo Alto, CA, USA). The sample preparation and the analysis procedure were carried out based on the procedure previous described (Zhang et al. 2020).

### Statistical analysis

All of experiments in this study were independently conducted at least three times. The data are represented as mean and standard deviation (± SD) calculated by the function STDEV in Microsoft Excel. Student’s *t* test was used for comparing the statistical difference among the groups of experiment data.

## Results

### Development and validation of MAMA-PCR

To verify the reliability of MAMA PCR in point mutation identification, γ-glutamyl kinase coding gene *proB* (encoding WT ProB) and proB* (encoding ProB^G149K^) harboring point mutation to relieve proline feedback inhibition in *C. glutamicum* ATCC 13032 were employed as amplification templates. In order to obtain the proB* template, we attempted to construct the strain *C. glutamicum* ATCC 13032proB^G149K^ by using CRISPR-Cas9 genome editing system (Peng et al. 2017). First, plasmid pFSC was transformed into strain *C. glutamicum* ATCC 13032 to obtain *C. glutamicum* ATCC 13032/pFSC. Then, 1-μg plasmid pFST-proB-proB^G149K^ carrying gRNA which recognized PAM site is located at the 149th amino acid encoded by *proB* and homologous repair template was transformed into *C. glutamicum* ATCC 13032/pFSC, and 66 transformants were obtained. Finally, we randomly selected six transformants for sequencing, of which four were *C. glutamicum* ATCC 13032proB^G149K^ strains and two were escaped cells without mutation. MAMA PCR depends on primer design, so we designed MAMA PCR primers whose bases at the 3′ terminus form a mismatch with the wild-type (WT) DNA template but match perfectly with the mutant DNA template (proB*). The 3′ terminal of the designed MAMA PCR primer is mismatched with WT*proB* template by 1 to 3 bases, respectively (Fig. 1b). Then, PCR reaction was carried out at primer annealing temperatures of 50, 54.2, 60.9, 67.8, and 70 °C respectively. As shown in Fig. 1c, the proB* and *proB* can be efficiently distinguished by raising the primer annealing temperature under the two and three base mismatches mediated by primer pair 15-AAG-F-2/R and 15-AAG-F-3/R. However, in the primer pair 15-AAG-F-1/R-mediated one-base mismatch system, the template *proB* and proB* cannot be distinguished by raising the primer annealing temperature. Even with the different primer pair 16-AGG-F/15-AGG-R, in which MAMA PCR primer 16-AGG-F has a single mismatch with the *proB* template, similar results can be obtained (Fig. 1d). These results suggest that increasing the number of 3′-terminal base mismatches and raising the primer annealing temperature of PCR can enhance the 3′ mismatch effect.

Considering the complexity of influencing factors in PCR amplification, we assume that different primer pairs have different amplification efficiency. Therefore, we replaced the other paired reverse primer, 17-AGG-R, which had the same Tm value as 15-AAG-R, without replacing the mismatched primer. We found that PCR mediated by primer pair 15-AAG-F-1/17-AGG-R can effectively identify single base mutation when the primer annealing temperature reaches 70 °C (Fig. 1e). Similarly, primer pair 16-AAG-F-1/17-AGG-R has similar results (Fig. 1e). These results indicate that adjusting primer pairs can also enhance the 3′ mismatch effect. In conclusion, our results suggested that the paired primers, the number of mismatched bases and primer annealing temperature can be adjusted to effectively identify whether there is point mutation in DNA template, even a single base substitution.

In order to test the accuracy of MAMA PCR in identifying point mutations, we used primer pair 15-AAG-F-1/17-AAG-R to carry out MAMA PCR on all 66 transformants. We found that 50 transformants had specific electrophoresis bands, including 4 transformants verified by sequencing, but 2 escaped cells failed to amplify bands (Figure S1). In addition, we can also effectively detect the point mutation in the previously constructed strains by using MAMA PCR (Figure S2). Next, we sequenced the remaining 60 transformants. As expected, all the transformants with bands had correct point mutations, while all the transformants without bands had no mutations (Table S1). These results have shown that MAMA PCR can accurately identify point mutations. MAMA PCR is a rapid and sensitive method for the detection of point mutations.

### Detection of zwf and gnd mutations by MAMA PCR

Increasing the availability of NADPH is commonly used to improve amino acid production by *C. glutamicu* (Hao et al. 2022; Li et al. 2023; Wu et al. 2019). It is reported that, the affinity of glucose-6-phosphate 1-dehydrogenase (Zwf) toward NADP + can be increased by mutating alanine at position 243th to threonine in *C. glutamicum* (Becker J 2007). In addition, the feedback inhibition of NADPH on 6-phosphogluconate dehydrogenase (Gnd) can be relieved by mutating threonine at position 361th into phenylalanine, which can improve the level of intracellular NADPH (Ohnishi J 2005). According to the genome editing method based on CRISPR, after Cas9/gRNA introduced double-strand breaks, the host was introduced point mutations into the genome through homologous recombination repair with homologous templates carrying damaged PAM or mutant seed sequences (Jiang et al. 2017; Peng et al. 2017). Therefore, if there is no suitable PAM at the target mutation site, it is difficult to precisely introduce point mutation at the target mutation site. In addition, the success of genome editing method based on CRISPR depends on the activity of gRNA (Creutzburg et al. 2020; Riesenberg et al. 2022). Thus, the precise introduction of point mutation by CRISPR method is limited to PAM position and gRNA activity. Based on CRISPR-Cas9 and CRISPR-Cas12a systems, there are three and two suitable PAM at the 243th site of Zwf and the 361th site of Gnd in *C. glutamicum*, respectively (Figure S3a). The results of gRNA activity test show that these gRNA activities are poor (Figure S3b). Therefore, the editing method based on CRISPR-Cas9/Cas12a is difficult to precisely introduce point mutation in the 243th of Zwf and the 361th of Gnd. However, the traditional double-crossover homologous recombination genome editing method based on pK18*mobsacB* is not limited by PAM and active gRNA. Therefore, here, we attempted to introduce Zwf^A243T^ and Gnd^S361F^ mutations into 13,032 genome by the double-crossover homologous recombination. Firstly, we constructed plasmids pK18-Zwf^A243T^ and pK18-Gnd^S361F^, which were transformed into 13,032 respectively, and obtained a primary recombinant strain with the whole plasmid integrated into the genome. Subsequently, the primary recombinant strain was cultured overnight in LBS medium, and then diluted and coated on LBS plate. For Zwf^A243T^ mutation, 47 recombinants were screened out, of which only 2 bands were specific by MAMA PCR (Fig. 2a). These two transformants were sequenced, and the results showed that point mutation indeed occur (Fig. 2c). Similarly, for Gnd^S361F^ mutation, 47 recombinants were screened, of which only 4 bands were specific by MAMA PCR (Fig. 2b). The sequencing results also showed that 4 recombinants had point mutations (Fig. 2d). In *C. glutamicum*, the editing method based on pK18*mobsacB* relies on random recombination, which will inevitably produce mutant strains. If it is mainly a revertant strain, it was necessary to sequence a large number of samples to detect point mutation recombinants. Therefore, MAMA PCR can screen mutant strains from low recombination events more quickly and at lower cost.

### Application of MAMA PCR in screening mutant library

In order to redirect metabolic flow to improve the accumulation of target metabolites, microbial metabolic network needs fine regulation. Generally speaking, optimizing key enzymes and fine-tuning gene transcription and translation are commonly used methods to regulate metabolic flow. However, in order to find the enzyme with the strongest performance and the optimal expression intensity of key node genes, it is an efficient strategy to construct mutant libraries of enzymes, promoters and RBS. In *C. glutamicum*, it is challenging to use traditional genome editing methods to construct efficient in situ mutation library. However, with the application of efficient CRISPR genome editing technology, the construction of in situ mutation library based on CRISPR method has been realized in *C. glutamicum* (Jiang et al. 2017; Liu et al. 2022a; Wang et al. 2021c). However, there are escape cells in both Cas12a-mediated and Cas9-mediated genome editing. On the other hand, the efficiency of gRNA that mediates the activities of Cas9 and Cas12a is different, which leads to more false-positive transformants in the constructed mutant library, which seriously affects the efficiency of screening mutants. Therefore, we try to screen the mutant transformants in the library by MAMA PCR, filter out false-positive transformants, improve the screening efficiency of mutants, and reduce the sequencing cost and the workload of mutant performance measurement.

**Fig. 3:**
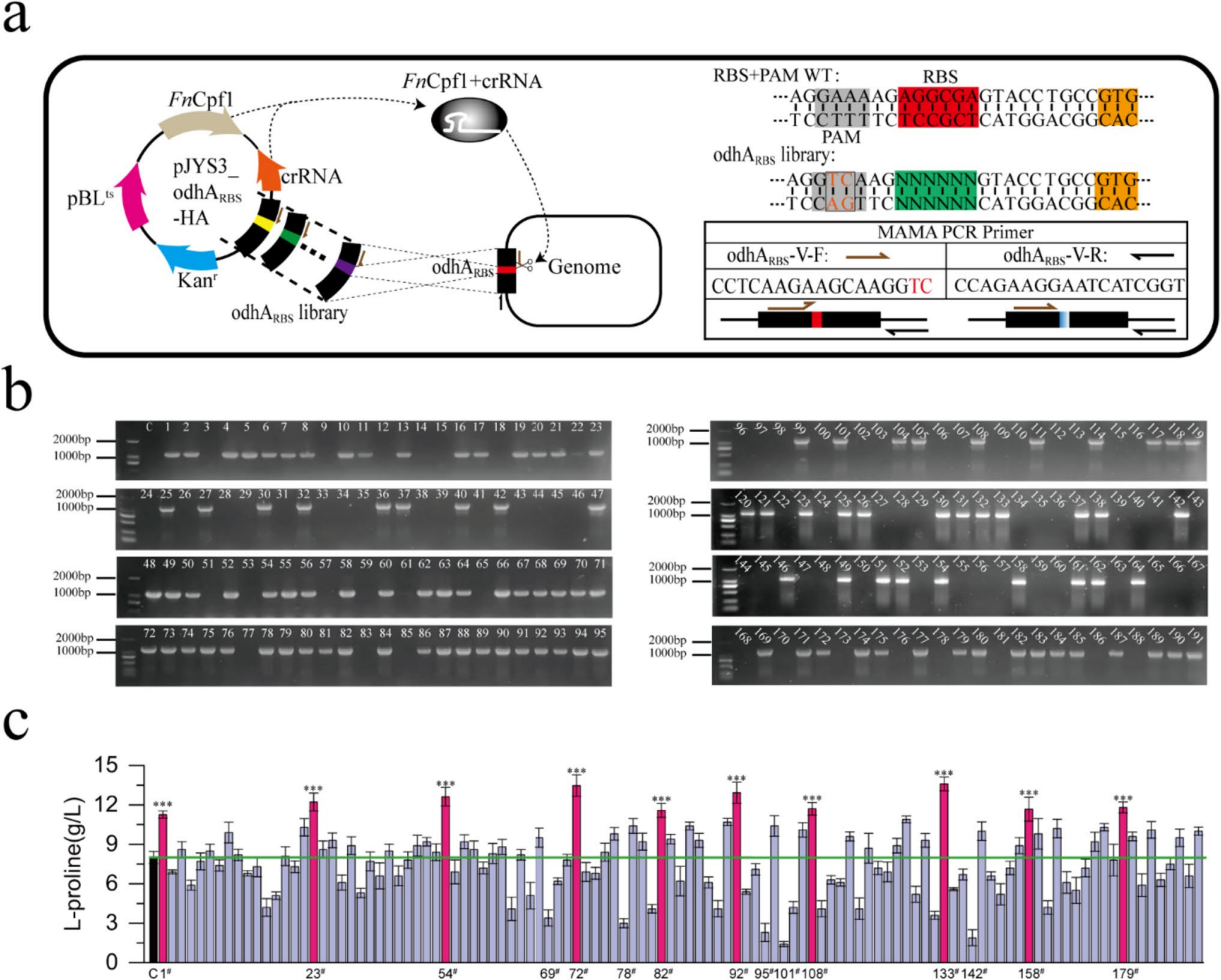
Application of MAMA PCR in screening mutant library. **a** Schematic diagram of construction and screening of saturated mutant library. Application of the pJYS3_odhA_RBS_-LIB plasmid library-based CRISPR-FnCpf1 system for RBS engineering in front of the *odhA* gene. Two bases of AA at PAM were mutated to TC for destroying PAM and designing MAMA PCR primers. The 3′ terminal of primer odhA_RBS_-V-F cannot adhere to the wild-type template, but can effectively adhere to the mutant template. The primer odhA_RBS_-V-R cannot be attached to the homologous arm. **b** MAMA PCR verification results of mutant library. Transformants with bands indicate that the genome has mutated. **c** The colonies were screened by 48-well plate fermentation and high-pressure liquid chromatography (HPLC) measurement of l-proline. The measurement results are shown in Table S3. Ten recombinants screened by 48-well fermentation exhibited significantly higher proline concentrations compared with the P12 strain (red bars). ***P < 0.001, Student’s two-tailed *t* test. Error bars indicate standard deviations from three parallel experiments

In our laboratory, a proline-producing strain *C. glutamicum* P12 (i.e., P12) was obtained through several rounds of chemical mutagenesis of *C. glutamicum* ATCC 13870, and it is required to supplement a large amount of l-glutamate to the fermentation medium for the efficient production of proline (Zhang 1996). When l-glutamate is not supplemented to the fermentation medium, proline accumulation decreases sharply (Figure S4). It is speculated that the supply of intracellular l-glutamate precursor is insufficient, which leads to the low proline accumulation when l-glutamate is not supplemented to the fermentation medium. Therefore, enhancing intracellular l-glutamate synthesis in P12 is beneficial to increase proline production. It is reported that weakening the expression of *odhA* can greatly increase the accumulation of l-glutamate (Wen and Bao 2019; Zhang et al. 2020). However, excessive inhibition of OdhA activity will impair cell growth; hence, fine-tuning *odhA* expression can give consideration to cell growth and l-glutamate synthesis. Therefore, based on CRISPR-Cas12a system, we constructed the RBS mutation library of *odhA* gene in situ (Fig. 3a). Every plasmid in the library pJYS3-odhA_RBS_-LIB contains the same crRNA targeting *odhA*_*RBS*_, and different plasmids harbor homologous repair templates with different RBS, which are transferred to strain P12. Because there are few transformants, we repeated three times of electro-transformation to obtain 191 transformants. We selected all the transformants and used MAMA PCR for preliminary screening. It was found that 112 transformants had RBS mutation, and the mutation efficiency was 58% (Fig. 3b). Therefore, 42% of false-positive transformants were filtered by MAMA PCR, which effectively reduced the workload of our subsequent fermentation test.

### Test of fermentation performance of library mutants

**Fig. 4:**
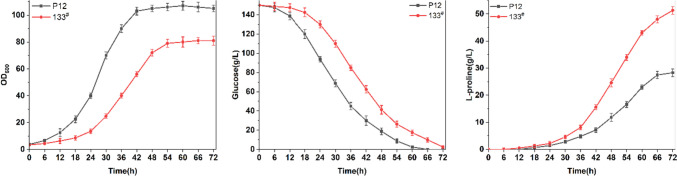
Determination of fermentation performance of proline-producing strain 133^#^ and P12 in shake flask. **a** Growth curves of strains 133^#^ and P12;** b** glucose consumption of strains 133^#^ and P12; **c** Proline accumulation of strains 133^#^ and P12. Error bars indicate standard deviations from three parallel experiments

Next, we tested the fermentation performance of 112 transformants by 48-well plate. l-Glutamate was not supplemented in the fermentation medium, and the fermentation was carried out for 72 h. According to the fermentation test, among 112 transformants, the proline production level is obviously different (Fig. 3c). P12 strain can only accumulate 8.1 ± 0.23 g/L proline in 48-well plate (Fig. 2c), which may be that the effect of dissolving oxygen in 48-well plate is worse than shake flask. However, 133^#^ transformant accumulated the highest proline of 13.60 ± 0.52 g/L and 101^#^ transformant accumulated the lowest proline of 1.42 ± 0.19 g/L (Fig. 3c). Compared with P12 strain, 10 transformants with increased proline production were selected for sequencing (Fig. 3c). The sequencing results are shown in Table 1. The results show that the RBS of all transformants tested have mutated. However, we failed to integrate a His-tagged *odhA* into the genome or insert a His-tag behind *odhA* in order to detect *odhA* expression by His-tag antibody, as previously reported (Zhang et al. 2020). Thus, the RBS Calculator (http://www.denovodna.com/software/dologin) was used to predict the translation initiation rate of RBS of *odhA* in the sequenced transformants. As shown in Table 1, the translation initiation rate of predicted RBS of *odhA* in all sequenced transformants is lower than wild-type RBS. Thus, it was speculated that the mutation of RBS of *odhA* in these sequenced transformants reduced the expression of *odhA*. Finally, we tested the proline fermentation of P12 strain and 133^#^ strain in shake flask with fermentation medium in which l-glutamate was not supplemented. Compared with P12 strain, the biomass of 133^#^ transformant decreased by 30% (Fig. 4a), and the sugar consumption rate was slower (Fig. 4b). However, after 72 h of fermentation, 133^#^ transformant could accumulate 51.3 ± 1.4 g/L proline (Fig. 4c), which was equivalent to the proline accumulation level of P12 strain in the medium supplemented with 30 g/L l-glutamate (Figure S4). Therefore, weakening the expression of *odhA* is an effective strategy to promote the synthesis of l-glutamate and amino acids with l-glutamate as precursor.

## Discussions

In this study, MAMA PCR was used to identify the point mutation ProB^G149K^ in *C. glutamicum*. The results suggested that the efficiency of MAMA PCR in identifying point mutations can be affected by replacing the paired primers of mismatched primers, raising the primer annealing temperature of PCR and increasing the number of mismatched bases at the 3′ terminal of MAMA primer. In addition, MAMA PCR can effectively improve the screening efficiency of RBS mutant library of *odhA* gene in *C. glutamicum*. By constructing RBS mutant library of *odhA*, the expression of *odhA* gene in proline-producer P12 was weakened, which promoted proline synthesis with l-glutamate as precursor (Table 2).

**Table 2:** The initial RBS and the replacement RBS of the *odhA* genes correspond to the predicted translation initiation rates

Name	Sequence (5′ → 3′)^*a*^	Predicted translation initiation rate (au)^*b*^
WT	AGAAGCAAGGAAAAGAGGCGAGTACCTGCC(GTG)	37.37
1^#^	AGAAGCAAGGTCAAGCGCTGAGTACCTGCC(GTG)	1.84
82^#^,158^#^	AGAAGCAAGGTCAAGAGTAGCGTACCTGCC(GTG)	1.51
108^#^,179^#^	AGAAGCAAGGTCAAGGTCAGTGTACCTGCC(GTG)	1.31
23^#^	AGAAGCAAGGTCAAGTTTTTTGTACCTGCC(GTG)	0.77
54^#^,92^#^	AGAAGCAAGGTCAAGATTATTGTACCTGCC(GTG)	0.31
72^#^,133^#^	AGAAGCAAGGTCAAGTACATTGTACCTGCC(GTG)	0.25

^*a*^Brackets represent the start codon sequence. ^*b*^The translation initiation rate calculation of the RBS sequences of *odhA* genes were performed using the RBS Calculator (http://www.denovodna.com/software/doLogin). Bold represents the SD sequence. Red markers represent base substitutions

PCR is a complex system, and the yield of PCR products is affected by many factors. Taq DNA polymerase without 3′-5′ exonucleolytic proofreading activity is required for MAMA PCR, but high-fidelity DNA polymerase with this activity is not suitable because it can cut off the mismatched base in the MAMA primer, allowing the modified primer to match with the template perfectly and effectively amplifying DNA. When MAMA PCR was carried out at lower primer annealing temperature, we observed that amplification with WT*proB* as template produced multiple PCR bands (Fig. 1c–f), which may be due to nonspecific bands caused by nonspecific amplification of MAMA PCR primers. It was report that the single internal mismatches in MAMA PCR primers will not significantly affect the product yield of PCR (Kwok et al. 1990). Under the background of genome, there are many positions with single internal mismatch with primers, so there will be multiple nonspecific PCR bands at low annealing temperature of primers. One of the possible explanations for why MAMA PCR can detect site mutation is that replacing the MAMA PCR primers can influence the quantity of PCR products. Due to primer mismatch, a pair of primers initially had a proper Tm value when they were fully attached to the DNA amplification template, but when they were attached to the mismatched DNA template, the Tm value between the mismatched primer and its paired primer increased, so that when the annealing temperature of PCR was increased, the mismatched primer could no longer be attached to the template at high temperature due to its low Tm value. As a result, the mismatched primer could not be used to initiate the PCR process. This also explains why, with the increase of PCR annealing temperature, when MAMA PCR primer uses mismatched DNA as template, the PCR yield is greatly reduced, while the mismatched template can be effectively amplified. Thus, setting the Tm value of the paired primer of the MAMA PCR primer higher than that of the MAMA PCR primer will assist to improve the sensitivity of MAMA PCR.

**Fig. 5:**
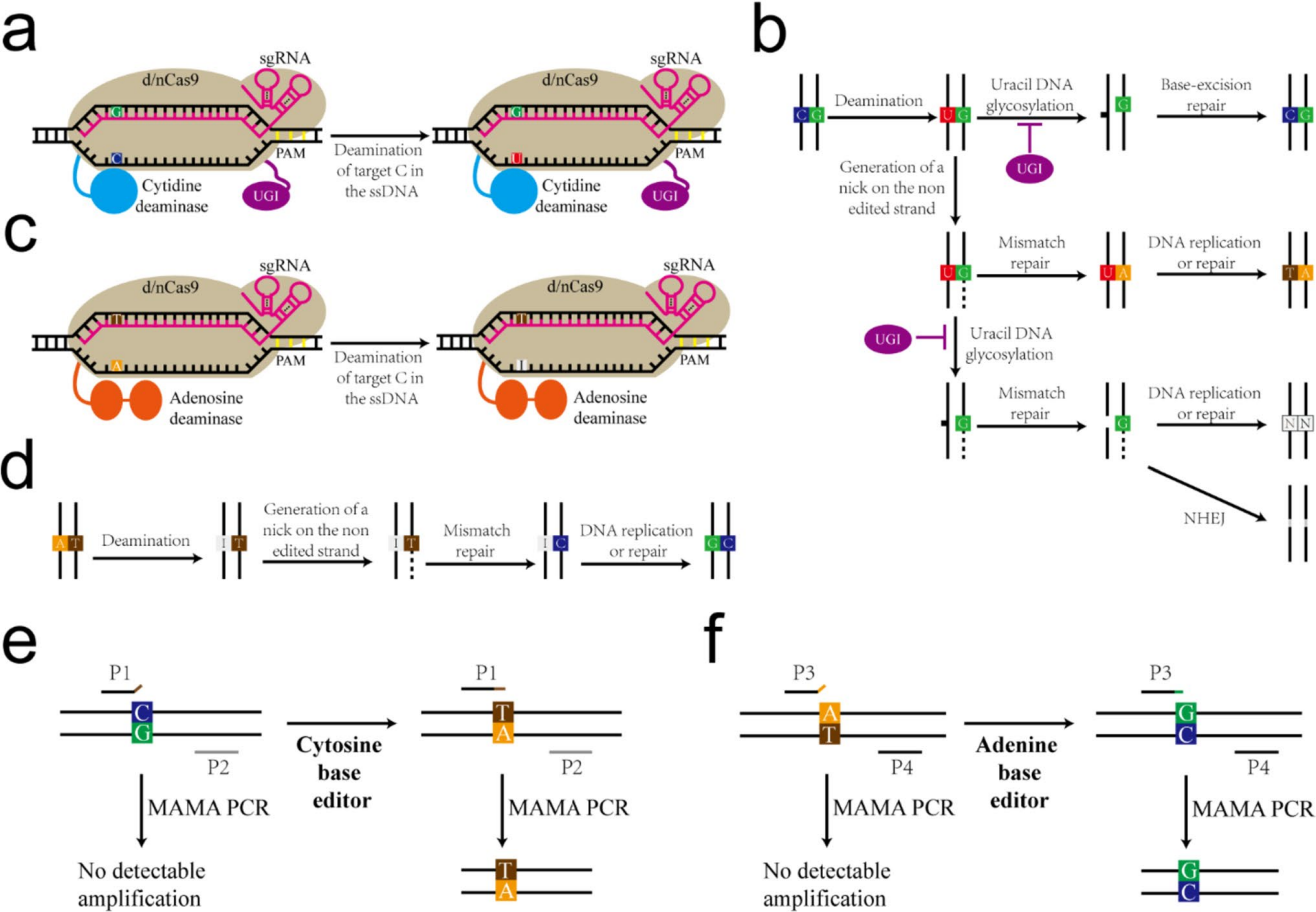
The application of MAMA PCR in base editing. **a** Cytosine base editor. **b** Cellular response to cytosine deamination. **c** Adenine base editor. **d** Cellular response to adenosine deamination. Abbreviations: d/nCas, nuclease-dead or nickase form of Cas protein; NHEJ, nonhomologous end joining; PAM, protospacer adjacent motif; ssDNA, single-stranded DNA; UGI, uracil DNA glycosylase inhibitor; **a–d** refers to previous reports (Wang et al. 2021a). **e** Application of MAMA PCR in cytosine base editing. **f** The application of MAMA PCR in adenine base editing

Although, we demonstrated the application of MAMA PCR in point mutation detection and mutation library screening in the conventional CRISPR genome editing technology. However, at present, based on CRISPR system, base editing that does not involve DNA double-strand breaks and does not need repair templates has been developed. The principle of base editing is that base editor deaminates the target site’s cytosine or adenine into uracil and hypoxanthine (Fig. 5a, c), which is finally converted from C to T or A to G by DNA replication or repair (Fig. 5b, d). Base editing is divided into cytosine editor and adenine editor. There may be four repair results after deamination of cytosine editor, but the inhibition of UGI can improve the conversion efficiency of C to A (Fig. 5b). The repair mechanism of A deamination on DNA is not as complicated as C deamination, which results in a high product purity (typically ≥ 99.9% A to G conversion) (Wang et al. 2021a). However, applications of ABEs in microorganisms suggest that their editing efficiencies are generally lower than those of CBEs (Luo et al. 2020; Tong et al. 2019; Wang et al. 2019a; Xin et al. 2019). Therefore, considering that base editing only involves base substitution, the complexity of repair products and low editing efficiency, MAMA PCR will be very suitable for screening transformants with correct base conversion after base editing (Fig. 5e, f). Although we take *C. glutamicum* as an example to show the application of MAMA PCR in point mutation identification and mutation library screening, this method will be applicable to other industrial microorganisms such as *Escherichia coli*, *Bacillus subtilis*, and yeast, which will be a powerful tool for screening mutants.

## Supplementary Information

Below is the link to the electronic supplementary material.

Supplementary file1 (PDF 1087 KB)

## Data Availability

The authors declare that all the data supporting the findings of this study are available within the paper, and its Supplementary Information is available from the corresponding author on request.
