# Supplementary material for: Rapid screening of point mutations by mismatch amplification mutation assay PCR
Source: Appl Microbiol Biotechnol. 2024 Feb 2;108(1):190. doi: 10.1007/s00253-024-13036-2 (PMC10837254; doi:10.1007/s00253-024-13036-2)
Supplement: Supplementary file 1 — Supplementary file1 (PDF 1087 KB) [file 253_2024_13036_MOESM1_ESM.pdf]

Supplementary Information

Rapid Screening of Point Mutations by Mismatch Amplification Mutation Assay PCR

Feng Zhang[1], Zhen Yang Liu[1], Shuai Liu[1], Wei Guo Zhang[1,*], Bing Bing Wang[1], Chang Lon Li[1], Jian Zhong Xu[1]

Supplementary Methods

Construction of plasmids and plasmid library.

For the construction of plasmid pFST-proB-proB^{G149K}, the fragment obtained by annealing primer sgRNA-proB-F/R was digested by *Eco*R I/*Hin*d III and cloned into pFST to obtain plasmid pFST-proB. The primers proB^{G149K}-F1/R1 and proB^{G149K}-F2/R2 were amplified by *C. glutamicum* ATCC 13032 genome to obtain fragments proB^{G149K}-1 and proB^{G149K}-2, respectively. The primer pFST-vector-F/R was amplified with pFST-proB as the template to obtain the fragment pFST-proB-vector; Finally, the three fragments were ligated by the ClonExpress MultiS One Step Cloning Kit to obtain plasmid pFST-proB-proB^{G149K}.

For the construction of plasmid pFST-gRNA1, the fragment obtained by annealing primer sgRNA-zwf-1-F/ sgRNA-proB-R was digested by *Eco*R I/*Hin*d III and cloned into pFST to obtain plasmid pFST-gRNA1.

For the construction of plasmid pFST-gRNA2, the fragment obtained by annealing primer sgRNA-zwf-2-F/ sgRNA-proB-R was digested by *Eco*R I/*Hin*d III and cloned into pFST to obtain plasmid pFST-gRNA2.

For the construction of plasmid pFST-gRNA4, the fragment obtained by annealing primer sgRNA-gnd-F/ sgRNA-proB-R was digested by *Eco*R I/*Hin*d III and cloned into pFST to obtain plasmid pFST-gRNA4.

For the construction of plasmid library pJYS3_0, the primer pJYS3_0-F/R was used to whole plasmid PCR using plasmid pJYS3_crtYf as template, and the PCR product was digested by *Dpn* I, and then purified. Then, the purified fragment was ligated by the ClonExpress II One Step Cloning Kit to obtain plasmid pJYS3_0.

For the construction of plasmid library pJYS3_gRNA3, the primer crRNA-zwf-F/R was used to whole plasmid PCR using plasmid pJYS3_crtYf as template, and the PCR product was digested by *Dpn* I, and then purified. Then, the purified fragment was ligated by the ClonExpress II One Step Cloning Kit to obtain plasmid pJYS3_gRNA3.

For the construction of plasmid library pJYS3_gRNA5, the primer crRNA-gnd-F/R was used to whole plasmid PCR using plasmid pJYS3_crtYf as template, and the PCR product was digested by *Dpn* I, and then purified. Then, the purified fragment was ligated by the ClonExpress II One Step Cloning Kit to obtain plasmid pJYS3_gRNA5.

For the construction of plasmid library pJYS3_odhARBS-LIB, the primer crRNA-odhARBS-F/R was used to whole plasmid PCR using plasmid pJYS3_crtYf as template, and the PCR product was digested by *Dpn* I, and then purified. Then, the purified fragment was ligated by the ClonExpress II One Step Cloning Kit to obtain plasmid pJYS3_odhA_{RBS}. The primer odhA_{RBS}-F/R was amplified by P12 genome to obtain the fragment odhA_{RBS}; The primer pJYS3-vector-F/R was amplified by using pJYS3-odhA_{RBS} as a template to obtain a fragment pJYS3-odhA_{RBS}-vector; Subsequently, the two fragments were ligated by the ClonExpress II One Step Cloning Kit to obtain plasmid pJYS3_odhA_{RBS}-HD_{odhA}. Then, the whole plasmid PCR was carried out by primer odhA_{RBS}-LIB-F/R with plasmid pJYS3_odhA_{RBS}-HD_{odhA} as template, and the PCR product was digested by *Dpn* I, and then purified. Finally, the purified fragment was ligated by the ClonExpress II One Step Cloning Kit and transformed into *E. coli* JM109 competent, and the plasmid library pJYS3_odhA_{RBS}-LIB was obtained.

For the construction of plasmid library pK18-Zwf^{A243T}, the upstream and downstream gene fragments of *zwf* were amplified from genomic DNA by primers Zwf^{A243T}-up-F/R and Zwf^{A243T}-down-F/R respectively, and then the two fragments were fused by overlapping extension PCR to obtain DNA

fragment Zwf^{A243T}-UD. Subsequently, Zwf^{A243T}-UD was inserted into *Hin*d III/*Xba* I-digested pK18*mobsacB* to obtain pK18-Zwf^{A243T}.

For the construction of plasmid library pK18-Gnd^{S361F}, the upstream and downstream gene fragments of *gnd* were amplified from genomic DNA by primers Gnd^{S361F}-up-F/R and Gnd^{S361F}-down-F/R respectively, and then the two fragments were fused by overlapping extension PCR to obtain DNA fragment Gnd^{S361F} -UD. Subsequently, Zwf^{A243T}-UD was inserted into *Xba* I/*Bam*H I-digested pK18*mobsacB* to obtain pK18-Zwf^{A243T}. DNA sequencing was used to verify the plasmids.

Supplementary Figures

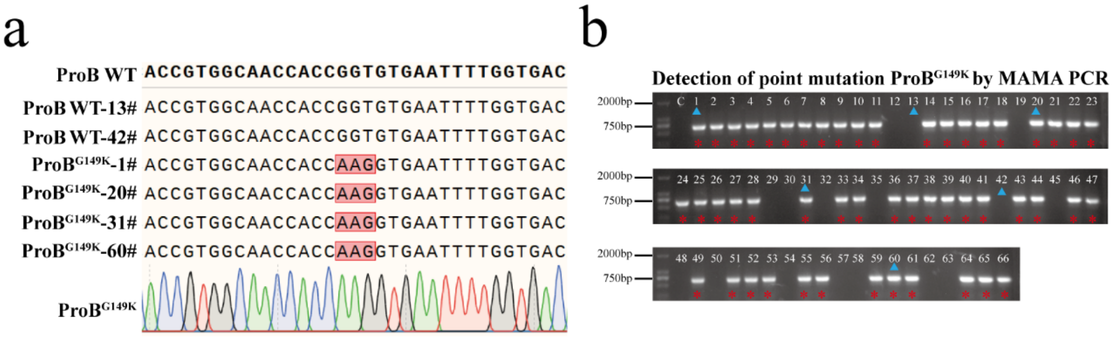

Figure S1. **Detection of point mutation ProB^{G149K} by MAMA PCR.** (**a**) Sequencing results of *proB* point mutation transformants edited by CRISPR-Cas9 system. Six transformants were randomly selected from 66 transformants for sequencing. GGT encodes glycine and AAG encodes lysine. (**b**)The mutation of ProB^{G149K} in 66 transformants was detected by MAMA PCR. Primers 15-AGG-F-1 (MAMA primer) and 17-AGG-R were used to detect ProB^{G149K} point mutation. The annealing temperature of primers is 70℃, and other PCR parameters are as described in Materials and Methods. * represent that the mutation was identified in the recombinant by MAMA PCR; ▲ represents the transformant selected for sequencing.

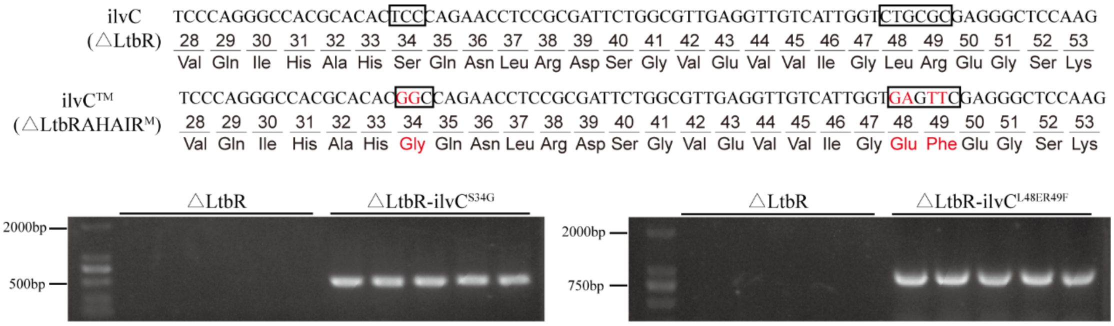

Figure S2. **Detection of point mutation *ilvC*^{TM} by MAMA PCR.** △LtbR and △LtbRAHAIR^{M} are leucine-producing bacteria, which are preserved in the laboratory(Wang et al. 2019). The extracted △LtbR and △LtbRAHAIRM genomes were used as templates for PCR amplification. Primers ilvC^{S34G}-V-F (MAMA primer) and ilvC^{S34G}-V-R were used to detect ilvC^{S34G} point mutation. The annealing temperature of primers is 66°C, and other PCR parameters are as described in Materials and Methods. Primers ilvC^{L48ER49F}-V-F (MAMA primer) and ilvC^{L48ER49F}-V-R were used to detect ilvC^{L48ER49F} point mutation. The annealing temperature of primers is 60.9°C, and other PCR parameters are as described in Materials and Methods.

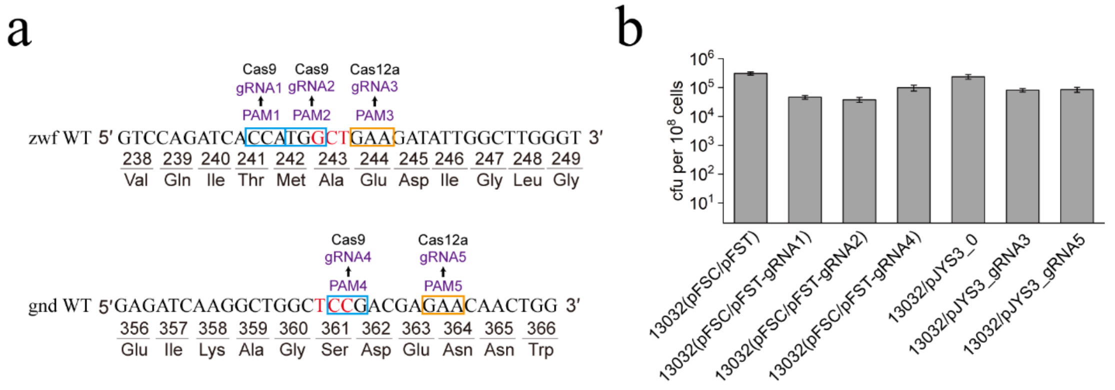

Figure S3. Activity determination of gRNA (guide RNA) at the mutation site of *zwf* and *gnd*. (a) PAM distribution of CRISPR-Cas9/Cas12a system near mutation *zwf* and *gnd*. The marked base in red corresponds to the amino acid residue to be mutated; gRNA1, gRNA 2 and gRNA 4 are sgRNA of Cas9 targeting PAM1, PAM2 and PAM4; gRNA 3 and gRNA 5 are crRNA of Cas12a targeting PAM 3 and PAM 5. **(b)** The activity of gRNA was detected in *Corynebacterium glutamicum* ATCC 13032 based on CRISPR-Cas9 and CRISPR-Cas12a. There are few transformants corresponding to gRNA, which indicates that gRNA activity is high, on the contrary, it indicates low activity. Error bars indicate standard deviations from three parallel experiments.

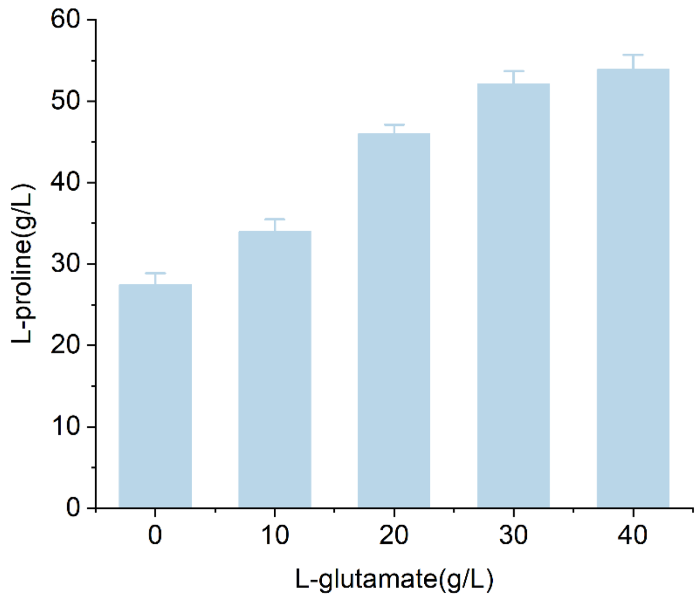

Figure S4. **Effect of L-glutamate addition on L-proline fermentation in P12 strain.** Error bars indicate standard deviations from three parallel experiments.

Supplementary Tables

Table S1. Sequencing results of *proB* site-directed mutation transformants edited by CRISPR-Cas9 system

**Strain**	**Sequence(5'→3')[a]**	**Strain**	**Sequence(5'→3')[a]**
**13032**	GCAACCACC**GGT**GTGAATTTT	**34#**	GCAACCACC**AAG**GTGAATTTT
**1#**	GCAACCACC**AAG**GTGAATTTT	**35#**	GCAACCACC**GGT**GTGAATTTT
**2#**	GCAACCACC**AAG**GTGAATTTT	**36#**	GCAACCACC**AAG**GTGAATTTT
**3#**	GCAACCACC**AAG**GTGAATTTT	**37#**	GCAACCACC**AAG**GTGAATTTT
**4#**	GCAACCACC**AAG**GTGAATTTT	**38#**	GCAACCACC**AAG**GTGAATTTT
**5#**	GCAACCACC**AAG**GTGAATTTT	**39#**	GCAACCACC**AAG**GTGAATTTT
**6#**	GCAACCACC**AAG**GTGAATTTT	**40#**	GCAACCACC**AAG**GTGAATTTT
**7#**	GCAACCACC**AAG**GTGAATTTT	**41#**	GCAACCACC**AAG**GTGAATTTT
**8#**	GCAACCACC**AAG**GTGAATTTT	**42#**	GCAACCACC**GGT**GTGAATTTT
**9#**	GCAACCACC**AAG**GTGAATTTT	**43#**	GCAACCACC**AAG**GTGAATTTT
**10#**	GCAACCACC**AAG**GTGAATTTT	**44#**	GCAACCACC**AAG**GTGAATTTT
**11#**	GCAACCACC**AAG**GTGAATTTT	**45#**	GCAACCACC**GGT**GTGAATTTT
**12#**	GCAACCACC**GGT**GTGAATTTT	**46#**	GCAACCACC**AAG**GTGAATTTT
**13#**	GCAACCACC**GGT**GTGAATTTT	**47#**	GCAACCACC**AAG**GTGAATTTT
**14#**	GCAACCACC**AAG**GTGAATTTT	**48#**	GCAACCACC**GGT**GTGAATTTT
**15#**	GCAACCACC**AAG**GTGAATTTT	**49#**	GCAACCACC**AAG**GTGAATTTT
**16#**	GCAACCACC**AAG**GTGAATTTT	**50#**	GCAACCACC**GGT**GTGAATTTT
**17#**	GCAACCACC**AAG**GTGAATTTT	**51#**	GCAACCACC**AAG**GTGAATTTT
**18#**	GCAACCACC**AAG**GTGAATTTT	**52#**	GCAACCACC**AAG**GTGAATTTT
**19#**	GCAACCACC**GGT**GTGAATTTT	**53#**	GCAACCACC**AAG**GTGAATTTT
**20#**	GCAACCACC**AAG**GTGAATTTT	**54#**	GCAACCACC**GGT**GTGAATTTT
**21#**	GCAACCACC**AAG**GTGAATTTT	**55#**	GCAACCACC**AAG**GTGAATTTT
**22#**	GCAACCACC**AAG**GTGAATTTT	**56#**	GCAACCACC**AAG**GTGAATTTT
**23#**	GCAACCACC**AAG**GTGAATTTT	**57#**	GCAACCACC**GGT**GTGAATTTT
**24#**	GCAACCACC**AAG**GTGAATTTT	**58#**	GCAACCACC**GGT**GTGAATTTT
**25#**	GCAACCACC**AAG**GTGAATTTT	**59#**	GCAACCACC**AAG**GTGAATTTT
**26#**	GCAACCACC**AAG**GTGAATTTT	**60#**	GCAACCACC**AAG**GTGAATTTT
**27#**	GCAACCACC**AAG**GTGAATTTT	**61#**	GCAACCACC**AAG**GTGAATTTT
**28#**	GCAACCACC**AAG**GTGAATTTT	**62#**	GCAACCACC**GGT**GTGAATTTT
**29#**	GCAACCACC**GGT**GTGAATTTT	**63#**	GCAACCACC**GGT**GTGAATTTT
**30#**	GCAACCACC**GGT**GTGAATTTT	**64#**	GCAACCACC**AAG**GTGAATTTT
**31#**	GCAACCACC**AAG**GTGAATTTT	**65#**	GCAACCACC**AAG**GTGAATTTT
**32#**	GCAACCACC**GGT**GTGAATTTT	**66#**	GCAACCACC**AAG**GTGAATTTT
**33#**	GCAACCACC**AAG**GTGAATTTT		

[a]The bold letters are the bases corresponding to the 149th amino acid of *proB*. Red represents base substitution.

Table S2. Primers used in this study

Primer	Sequence (5'→3') [a]
sgRNA-proB-F	ggaattcaatgacacgtggcaaccacgttttagagctagaaatagcaagttaaaataaggctagtcc
sgRNA-proB-R	cccaagcttaaaaaagcaccgactcggtgccactttttcaagttgataacggactagccttattttaact
pFST-vector-F	tctgatcaagagacaggat
pFST-vector-R	tcttgatcccctgcgccat
proB^{G149K}-F1	atggcgcaggggatcaagacctacagccaggaagcaa
proB^{G149K}-R1	attcaccttggtggttgccacggtgtcattttcattgacga
proB^{G149K}-F2	tggcaaccaccaaggtgaattttggtgacaacgac
proB^{G149K}-R2	atcctgtctcttgatcagacccaagatctccacgatc
pJYS3_0-F	agcctttcgcctatccagcagtcttcct
pJYS3_0-R	ctggataggcgaaaggctcagtcgaaag
sgRNA-zwf-1-F	ggaattccaagccaatatcttcagccgttttagagctagaaatagcaagttaaaataaggctagtcc
sgRNA-zwf-2-F	ggaattctgaccacgtccagatcaccagttttagagctagaaatagcaagttaaaataaggctagtcc
sgRNA-gnd-F	ggaattcaacgtcccagttgttctcgtgttttagagctagaaatagcaagttaaaataaggctagtcc
crRNA-zwf-F	ccatggtgatctggacgtggtcaaataaaacgaaaggctc
crRNA-zwf-R	acgtccagatcaccatggctatctacaacagtagaaattc
crRNA-gnd-F	tcggagccagccttgatctcgaaataaaacgaaaggctc
crRNA-gnd-R	agatcaaggctggctccgacgaatctacaacagtagaaattc
crRNA-odhA$_{RBS}$-F	cttgcttcttgagggtttattgagaaataaaacgaaaggctc
crRNA-odhA$_{RBS}$-R	ctcaataaaccctcaagaagcaagatctacaacagtagaaattc
pJYS3-vector-F	ctagattgacagctagctcagt
pJYS3-vector-R	ccggtgaacagttgttctact
odhA$_{RBS}$-F	gtagaacaactgttcaccgggtacatgggcctgatgtt
odhA$_{RBS}$-R	gagctagctgtcaatctaggatcgtaggtggaggtgat
odhA$_{RBS}$-LIB-F	cctcaagaagcaaggtcaagNNNNNNgtacctgccgtgagcagc
odhA$_{RBS}$-LIB-R	cttgaccttgcttcttgagggtttattgagc
Zwf^{A243T}-up-F	gctctagacaaacaccgtcaacaccat
Zwf^{A243T}-up-R	catggtgatctggacgtggt
Zwf^{A243T}-down-F	accacgtccagatcaccatgaccgaagatattggcttgggtgg
Zwf^{A243T}-down-R	cccaagcttgtggactcggtaactgcag
Gnd^{S361F}-up-F	cgggatccgcaacactgtcgctgtct
Gnd^{S361F}-up-R	gttctcgtcaaagccagccttgatctcgtc
Gnd^{S361F}-down-F	ggctggctttgacgagaacaactgggacg
Gnd^{S361F}-down-R	gctctagagggatttaccggcgctat
15-AAG-F1	ccgtggcaaccacc**a**
15-AAG-F2	cgtggcaaccacc**aa**
15-AAG-F3	gtggcaaccacc**aag**
16-AAG-F1	accgtggcaaccacc**a**
16-AAG-F2	ccgtggcaaccacc**aa**
16-AAG-F3	cgtggcaaccacc**aag**
15-AAG-R	gttgatgcggtcggcg
17-AAG-R	tagacaggttgatgcgg

ilvC^{S34G}-V-F	aatcgcggaggttctgg**cc**
ilvC^{S34G}-V-R	aacatcttccgcgcccgag
ilvC^{L48ER49F}-V-F	ttggagccctcg**aa**c**tc**
ilvC^{L48ER49F}-V-R	ccacatcctgtccgtact
Zwf^{A243T}-V-F	ccagatcaccatg**a**c**c**
Zwf^{A243T}-V-R	gccaaccaccaaaatgatc
Gnd^{S361F}-V-F	ccagttgttctcgtc**aa**
Gnd^{S361F}-V-R	ggagataatctcgcacaga
odhA$_{RBS}$-V-F	cctcaagaagcaagg**tc**
odhA$_{RBS}$-V-R	ccagaaggaatcatcggt

[a]The bold letters in red represent the mismatched bases on the MAMA PCR primer with the unmutated template.

Table S3. L-proline fermentation of transformants screened by MAMA PCR from the RBS engineering library

**Transformants**	**L-proline (g/L)**	**Transformants**	**L-proline (g/L)**
P12	8.04±0.46	86#	6.24±1.12
1#	11.26±0.28	87#	10.41±0.30
2#	6.93±0.15	88#	9.36±0.54
4#	8.64±0.60	89#	6.18±0.42
5#	5.92±0.38	90	4.15±0.62
6#	7.71±0.64	91#	10.72±0.27
7#	8.55±0.51	92#	12.93±0.80
8#	7.42±0.43	93#	5.45±0.19
10#	9.93±0.79	94#	7.18±0.43
11#	8.28±0.43	95#	2.37±0.69
13#	6.75±0.21	99#	10.46±0.78
16#	7.29±0.76	101#	1.42±0.19
17#	4.18±0.67	104#	4.20±0.45
19#	5.11±0.29	105#	10.12±0.55
20#	8.14±0.71	108#	11.70±0.48
21#	7.26±0.44	111#	4.15±0.61
22#	10.27±0.66	114#	6.36±0.32
23#	12.22±0.68	117#	6.24±0.28
25#	8.59±0.65	118#	9.43±0.35
27#	9.31±0.56	119#	4.07±0.82
30#	6.09±0.57	120#	8.37±1.13
32#	8.76±0.68	121#	7.20±0.5
36#	5.32±0.35	123#	6.89±0.75
37#	7.71±0.72	125#	8.91±0.55
40#	6.64±0.99	126#	10.86±0.25
42#	8.55±0.52	130#	5.19±0.61
47#	6.66±0.76	131#	9.28±0.52
48#	7.87±0.63	132#	3.59±0.31
49#	8.94±0.79	133#	13.60±0.52
50#	9.26±0.32	137#	5.56±0.12
52#	8.43±0.64	138#	6.72±0.41
54#	12.62±0.71	142#	1.91±0.61
55#	6.91±0.91	146#	10.04±0.71
56#	9.15±0.52	149#	6.64±0.31
58#	8.58±0.65	151#	5.25±0.81
60#	7.22±0.45	152#	7.21±0.51
62#	8.33±0.77	154#	8.90±0.61
63#	8.80±0.58	158#	11.67±0.91

64#	4.12±0.89	161#	9.82±1.16
66#	8.26±0.40	162#	4.17±0.51
67#	5.09±1.01	164#	10.21±0.71
68#	9.47±0.73	169#	6.12±0.82
69#	3.35±0.61	171#	5.54±0.99
70#	6.18±0.25	172#	7.19±0.67
71#	7.79±0.44	174#	9.16±0.72
72#	13.47±0.81	175#	10.27±0.28
73#	6.93±0.72	177#	7.80±1.21
74#	6.77±0.45	179#	11.81±0.41
75#	8.40±0.69	180#	9.62±0.33
76#	9.81±0.48	182#	5.88±0.86
78#	3.04±0.34	183#	10.09±0.63
79#	10.42±0.56	184#	6.32±0.5
80#	9.21±0.65	185#	7.51±0.43
81#	4.13±0.32	187#	9.49±0.66
82#	11.59±0.53	189#	6.60±0.88
84#	9.40±0.36	191#	10.03±0.32

References

Wang YY, Zhang F, Xu JZ, Zhang WG, Chen XL, Liu LM (2019) Improvement of l-Leucine Production in *Corynebacterium glutamicum* by Altering the Redox Flux. Int J Mol Sci 20(8):2020. doi:10.3390/ijms20082020
